# Patient-related factors associated with an increased risk of being a reported case of preventable harm in first-line health care: a case-control study

**DOI:** 10.1186/s12875-020-1087-4

**Published:** 2020-01-29

**Authors:** Rita Fernholm, Martin J. Holzmann, Caroline Wachtler, Robert Szulkin, Axel C. Carlsson, Karin Pukk Härenstam

**Affiliations:** 1 Division of Family Medicine and Primary Care, Department of Neurobiology, Care Sciences and Society, Karolinska Institutet, Alfred Nobels allé 23, D2, S-141 83 Huddinge, Sweden; 2 Department of Medicine, Solna, Karolinska Institutet, SE-171 71 Stockholm, Sweden; 3 Functional Area of Emergency Medicine, Karolinska University Hospital, Huddinge, SE-141 86 Stockholm, Sweden; 4 Scandinavian Development Services, Danderyd, Sweden; 5 Department of Learning, Informatics, Management and Ethics, Medical Management Centre, Karolinska Institutet, SE-171 77 Stockholm, Sweden

**Keywords:** Primary health care, Emergency medical services, Emergency care, Medical errors, Mental health disorders, Psychiatric illness, Patient harm, Preventable harm, Adverse Events

## Abstract

**Background:**

Patient safety issues in primary health care and in emergency departments have not been as thoroughly explored as patient safety issues in the hospital setting. Knowledge is particularly sparse regarding which patients have a higher risk of harm in these settings. The objective was to evaluate which patient-related factors were associated with risk of harm in patients with reports of safety incidents.

**Methods:**

A case–control study performed in primary health care and emergency departments in Sweden. In total, 4536 patients (cases) and 44,949 controls were included in this study. Cases included patients with reported preventable harm in primary health care and emergency departments from January 1st, 2011 until December 31st, 2016.

**Results:**

Psychiatric disease, including all psychiatric diagnoses regardless of severity, nearly doubled the risk of being a reported case of preventable harm (odds ratio, 1.96; *p* < 0.001). Adjusted for income and education there was still an increased risk (odds ratio, 1.69; p < 0.001). The preventable harm in this group was to 46% diagnostic errors of somatic disease.

**Conclusion:**

Patients with psychiatric illness are at higher risk of preventable harm in primary care and the emergency department. Therefore, this group needs extra attention to prevent harm.

## Background

Approximately 40 million people are harmed in health care worldwide every year [1]. About 5 to 8% of all hospital admissions in high-income countries result in harm that could have been prevented [2–4]. Patient safety and preventable harm to patients in first-line health care, defined as emergency care and primary health care (PHC), is a rising issue because first-line health care represents the largest volume of health care encounters [5]. The financial and economic costs of safety lapses in primary and other ambulatory care are high, about 2.5% of total health expenditure [6].

Several types of preventable harm have been identified: diagnostic errors; prescribing, dispensing, and administering medication; and incomplete transfer of information across care boundaries [7]. In first-line health care, diagnostic error—defined as delayed, missed, or incorrect diagnoses [8] —is a common type among serious preventable harm [9–11]. In these cases, patients do not receive the correct treatment in a timely manner. The frequency of diagnostic errors in outpatient care has been estimated at 5% [12]. Both PHC and emergency departments (EDs) are often stressful environments with high rates of diagnostic decision-making. Another type of harm in first-line health care is medication error, which occurs at a rate of 3 to 10% [13]. Harm of treatment that does not include medication is low in this setting.

Many health care-related factors that increase the risk of preventable harm have been identified, but only a few patient-related risk factors for harm are known. Comorbidities can increase the risk of harm in hospitalised patients according to evidence from patients with metastatic cancer, coagulopathies, fluid/electrolyte disorders, or serious mental illness [14, 15]. Patients of advanced age and patients with many medications have a higher risk of harm [16, 17]. In PHC, some groups are at higher risk of adverse safety events. A systematic review from 2018 showed sex and ethnic disparities in risks of adverse safety events [18]. However, studies on the association between disparities in income and educational level and preventable harm are sparse. Knowledge of patient-related risk factors in first-line health care is limited. Reports of safety incidents and claims do not show the true incidence of harm but can, if the material is large enough, yield information about the safety of the system.

On the basis of previous findings in smaller studies, we hypothesised that patient characteristics such as socioeconomic status, having foreign background, or psychiatric illness could affect the risk of harm in first-line health care. We therefore evaluated which patient-related factors were associated with risk of harm in patients with reports of safety incidents.

## Methods

### Study design

A case–control study of patient-related factors associated with risk of preventable harm in first-line health care.

### Setting

The study used data collected in Sweden from January 1st, 2011 until December 31st, 2016. The setting was PHC and emergency departments. It can be argued that PHC and ED are different settings, but both these settings represent the first contact with healthcare for patients experiencing new symptoms and both contexts have a high density of diagnostic decision making.

### Databases

The two databases used are also described in previous work by the researchers [19]. The first database was the voluntary nationwide patient-reported harm database. In Sweden, preventable harm, such as delayed diagnosis leading to harm or harm of treatment leading to hospitalisation or sick-leave, is compensated by a nationwide non-punitive malpractice carrier and insurance company called Landstingens Ömsesidiga Försäkringsbolag (LÖF). In Sweden, patient malpractice claims are handled administratively and compensated if an independent review confirms patient injury resulting from medical error. Claims data included type and nature of injury, affected body part(s), diagnoses and procedure codes, as well as information on region, hospital, department, patient age and gender. All cases of preventable harm, from the whole country, from primary health care and from the ED during the years of 2011 throughout 2016 where included. Compensated cases in this database have a mortality rate of approximately 3% as a direct or indirect consequence of the safety incident. The severity of the preventable harm is evaluated in the same standardised way from PHC and ED, in six levels, sick leave < 3 months, sick leave > 3 months, disability 1–15%, disability 16–30%, disability > 30% and death.

The second database was the mandatory nationwide safety-incident database of health care-facility-reported serious safety incidents, including serious preventable harm or a risk of serious preventable harm. In the database ‘serious’ is defined as a patient safety risk that could lead to long-lasting non-negligible damage, to the patient needing significantly increased care, or to the patient’s death. Reported cases in this database are often more serious than the patient reported database, with a mortality of approximately 28% as a consequence of the safety incident. Even if reporting is mandatory, there are probably serious safety incidents that are not reported. We used the database with all cases, from the whole country, from primary care during 2011 throughout 2016.

### Inclusion criteria

We included all cases reported by patients that had experienced preventable harm, in PHC or EDs. From the health care-reported cases, only primary care was included because the cases were in paper form and it was very labour intense to analyse the material and digitise it. The reports which the Health and Social Care Inspectorate assessed as ‘satisfactorily investigated’ during the study period were included in this study.

### Exclusion criteria

Swedish residents have a unique personal identification number and this number was used to enable accurate linkage of national health care registers with the cases included from the databases. Cases with missing personal identification numbers were excluded (*n* = 38). Cases that were assessed by the research team as non-preventable suicides, were also excluded (*n* = 96). A non-preventable suicide was defined as that in which the patient had not contacted a health care provider the 4 weeks prior to his or her death.

### Controls

We matched each case to 10 controls. The controls were matched for age, sex and residential area (*n* = 44,949 individuals). The residential areas in Sweden are small, each usually comprising < 1000 persons. Statistics Sweden provided the controls and performed the matching procedure based on each individual’s personal identification number. Statistics Sweden also provided information about each person’s foreign background, education, and income. Furthermore, they matched the information provided by the National Board of Health and Welfare to all individuals. That information included discharge information from all hospital admission and reported psychiatric illness.

### Measurement

Diagnoses were classified according to the International Classification of Diseases, 10th revision (ICD-10). In Sweden, a visit to a public health care facility always results in at least one diagnosis from the ICD-10, either a specific diagnosis or a descriptive diagnosis of the symptoms. A patient can receive several diagnoses during a visit, and all diagnoses were used in the study. A psychiatric diagnosis received at any time during the 3 years preceding the date of the reported preventable harm (for cases) or the matching date (for controls) was used as an indication of psychiatric disease. Both acute and chronic psychiatric diagnosis were included. There is no nationwide registry for the diagnoses from primary health care, so the diagnoses are from hospital and specialised ambulatory care including psychiatric outpatient clinics. Psychiatric illness was defined as ICD-10 codes F01.0 to F99.9. That includes all psychiatric diagnosis used in the Swedish health care. For example, ICD F32.0-F41.9 indicates depression or anxiety while F10.0-F19.9 is alcohol and substance related psychiatric disease.

The socioeconomic status was evaluated by the education level and individual disposable income. Education level was divided into four levels: level 1, up to 9 years of school; level 2, 11 to 12 years of school; level 3, bachelor and master’s degrees; and level 4, postgraduate education. Income was assessed using individual disposable income, divided into quartiles.

Foreign background was divided into four categories: both parents from Sweden, one parent from Sweden, no parent from Sweden, and the person herself/himself born outside of Sweden.

### Statistical analyses

The baseline characteristics of the safety incidents among cases and controls, respectively, are presented using descriptive statistics: continuous variables are summarised as mean and standard deviation and categorical variables as frequency and percentage.

The association between patient-related factors and the risk of a reported patient safety event was estimated by odds ratios (ORs), using conditional logistic regression models for matched case–control data [20]. In addition, 95% confidence intervals (CIs) for the ORs are presented, and group differences were tested using the Wald test with a 5% significance level. Results from both crude (unadjusted) models and multivariate models (adjusting for income and education) are presented. The subgroup of patients with diagnoses related to alcohol and drug intake was tested separately with the same statistical methods. To assess whether the effect of psychiatric illness on the risk of preventable harm is different in primary care and emergency care respectively, we fitted a conditional logistic regression model with an interaction between psychiatric illness status and an indicator variable of type of care (primary or emergency). All analyses were conducted using Stata 14.2 (StataCorp, College Station, TX, USA).

## Results

### Sample characteristics

**Fig. 1:**
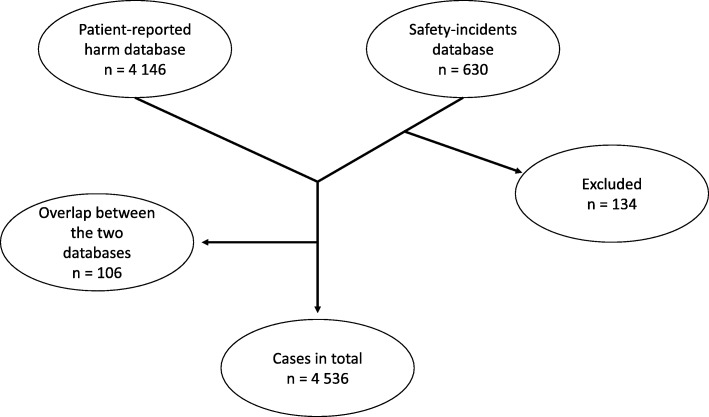
Cases included in the study

In total, 4536 patients (cases) (see Fig. 1) and 44,949 controls were included in this study (10 controls per case). Their characteristics are shown in Table 1. There were slightly more women than men (57% vs. 43%, respectively). The cases had a higher degree of comorbidities (cardiovascular disease, psychiatric disease and cancer). The controls were matched for age, sex, and residential area.

**Table 1:** Baseline characteristics for cases (patients with preventable harm) and matched population controls

Variable	Cases^a^	Controls^b^
Number of participants (n)	4536	44,949
Female (%)	57	57
Age, mean (SD)	49 (21)	49 (21)
Age, range (yrs.)	0–98	0–98
Cancer, n (%) ^b^	236 (5.2)	1573 (3.5)
Cardiovascular disease, n (%) ^b^	590 (13)	3326 (7.4)
Psychiatric diagnosis, n (%) ^b^	430 (9.5)	2248 (5.0)
Preventable harm reported in primary care, n (%)	3292 (73)	–
Preventable harm reported in emergency care, n (%)	1244 (27)	–
Psychiatric diagnoses, excluding those related to alcohol and drugs, n (%)	313 (6.9)	1809 (4.0)
Psychiatric diagnoses, only those related to alcohol and drugs, n (%)	117 (2.6)	447 (1.0)
Disposable income		
Lowest quartile, n (%)	1024 (24)	10,507 (25)
2nd quartile, n (%)	1213 (29)	10,320 (25)
3rd quartile, n (%)	1076 (25)	10,444 (25)
4th quartile, n (%)	921 (22)	10,605 (25)
Education level		
Elementary school or less (up to 9 years of school), n(%)	933 (22)	9547 (23)
Secondary school (11 to 12 years of school), n (%)	1993 (48)	18,509 (45)
Bachelor’s or master’s degree, n (%)	1235 (30)	12,621 (31)
Post graduate education	19 (0.45)	381 (0.93)
Foreign born vs born in Sweden, n (%)	624 (14)	7117 (16)
Parental country of birth		
Two Swedish-born parents	3403 (75)	33,847 (75)
One foreign-born parent	314 (6.9)	2629 (5.8)
Two foreign-born parents	189 (4.2)	1356 (3.0)

^a^ Mean values with standard deviation (SD) for continuous variables and n (%) for categorical variables. All cases were matched for age, sex and residential area

^b^ Cancer ICD-10 codes C01.9-C97.9, Cardiovascular disease ICD-10 codes I01.0-I99.9, Psychiatric disease F01.0-F99.9

### Psychiatric illness

Risk factors for preventable harm, assessed by conditional logistic regression, are shown in Table 2. Psychiatric disease nearly doubled the risk of preventable harm in first-line health care (OR, 1.96; *p* < 0.001). The interaction between psychiatric illness and the indicator of type of care (primary or emergency) was highly non-significant, *p* = 0.83. Thus, we concluded that the association between psychiatric illness and the risk of preventable harm was equivalent for emergency care and primary care patients. The preventable harm was mostly somatic harm as oppose to psychiatric harm/suicide, primarily involving diagnostic errors of somatic disease (46% of all preventable harm in this category was due to diagnostic errors, the smaller categories were harm from falls, surgical complications, medication-related and cross infections). Adjusted for income and education there was still an increased risk (odds ratio, 1.69; *p* < 0.001).

**Table 2:** Association of different risk factors and preventable harm (conditional logistic regression)

	Crude model, odds ratio (95% CI)	*p*-value	Adjusted for income and education	*p*-value
No psychiatric diagnoses	1 (Ref)		1 (Ref)	
Psychiatric diagnoses	1.96 (1.76–2.19)	< 0.001	1.69 (1.73–2.13)	< 0.001
Disposable income				
Lowest quartile	1 (Ref)		1 (Ref)	
2nd quartile	1.23 (1.12–1.36)	< 0.001	1.18 (1.07–1.31)	0.001
3rd quartile	1.05 (0.95–1.16)	0.36	1.04 (0.94–1.15)	0.47
4th quartile	0.86 (0.77–0.96)	0.006	0.87 (0.78–0.98)	0.02
Education level				
Elementary school or less (up to 9 years of school)	1 (Ref)		1 (Ref)	
Secondary school (11 to 12 years of school)	1.11 (1.01–1.21)	0.02	1.13 (1.03–1.24)	0.008
Bachelor’s or master’s degree	1.00 (0.91–1.11)	0.93	1.08 (0.97–1.20)	0.14
Post graduate education	0.51 (0.32–0.82)	0.005	0.59 (0.37–0.95)	0.03
Origin				
Two Swedish-born parents	1 (Ref)		1 (Ref)	
Born outside of Sweden	0.86 (0.79–0.95)	0.002	0.91 (0.82–1.00)	0.05
Born in Sweden with one foreign-born parent	1.21 (1.07–1.37)	0.003	1.18 (1.03–1.35)	0.02
Born in Sweden with two foreign-born parents	1.41 (1.20–1.67)	< 0.001	1.26 (1.04–1.53)	0.02

All cases were matched for age, sex, and residential area. The crude model was unadjusted. The adjusted model was adjusted for disposable income and four levels of education

The most common psychiatric diagnoses were depression and anxiety (*n* = 160), alcohol- and drug-related psychiatric disorders (*n* = 104), dementia (*n* = 27), bipolar disease (*n* = 24), and psychotic disorders (*n* = 20).

### Socioeconomic factors

Differences in income and education had some impact on the risk of preventable harm (Table 2). The risk in the highest income group (highest quartile) was slightly lower (OR, 0.86; *p* < 0.01) than that in the lowest quartile. The highest educational level (postgraduate) had a lower risk (OR, 0.51; p < 0.01) than the education level of ≤9 years of school. The group postgraduate patients with harm was small with only 19 cases why all results in this group should be interpreted cautiously.

### Foreign background

Being born in another country was associated with a somewhat lower risk of being a reported case than being born in Sweden by Swedish-born parents. Two parents of foreign origin also increased this risk (OR, 1.41; *p* < 0.001) (see Table 2).

## Discussion

### Main findings

We found that patients with a psychiatric diagnosis had a nearly two-fold higher risk of being a reported case of preventable harm in first-line health care. The most common type of harm was diagnostic errors and less common were suicide, medication error or harm by treatment.

The effects of income, education level, and foreign background were modest. That might reflect the fact that Sweden is a land of smaller inequality in terms of income and education than many other countries, making differences in risk more difficult to study. The slight increase in risk when born with two parents of foreign origin could indicate that this group does not receive the same care as other Swedes, for unknown reasons.

This has not been explored earlier in primary care, with the range of all types of psychiatric illness.

### Strengths and limitations

This study has limitations, including unreported cases and the lack of a prospective design. PHC and ED have been merged in this study. It can be seen as a limitation, but both these settings represent the first contact with healthcare for patients experiencing new symptoms.

A major limitation is that the diagnoses used are diagnoses from hospital and specialised ambulatory care, lacking diagnoses from primary care. Data on primary care diagnoses do not exist on a national level in Sweden and could therefore not be included. The national registry of diagnoses has a coverage of 99% of all diagnoses from hospitals during the years of this study and 96% of all diagnoses from out-patient clinics. This fact could result in an underrepresentation of mild psychiatric disease posing a bias. Furthermore, bias toward more serious cases of harm may have existed because of a threshold to report and because of cases that were not reported, and which most likely were less severe. Incident reports and malpractice are not methods that capture the true incidence of harm but since patients with psychiatric disease probably report harm to a lesser extent than others, based on the fact that they seek health care later [21], the findings may be of interest. We were unable to evaluate whether any age group or sex is at higher risk of harm because this was a case–control study in which controls were matched for age and sex. Earlier studies have shown slight overrepresentation of claims from women [22]. Patients with psychiatric illness have a higher mortality of somatic diseases than do patients without psychiatric illness [23]. This could result in a higher risk of health care-related harm associated with comorbidities rather than psychiatric illness in itself. Additionally, harm in the form of missed and delayed diagnoses among patients with psychiatric illnesses could increase their mortality.

Finally, a limitation is that we did not have access to data on social problems and social diseases that could have confounded our results. There might be other factors influencing the results, but the data is all reported cases from the whole country with matched controls which mitigate the risk of effect of geographical area.

The main strength of this study is that it was based on nationwide data and included a relatively large sample of 4536 patients from two complementary sources. Another major strength is that the Swedish registers enabled us to find matched controls. Moreover, this study included cases from both PHC and emergency care and a broad range of psychiatric illness, not only serious mental illness. Most prior studies were conducted in hospital settings and with patients with schizophrenia or other serious mental health problems. Furthermore, Sweden has a no-blame insurance system that compensates patients for injuries that result from errors in medical practice, which can facilitate reporting.

### Comparison with previous studies

Several large studies have confirmed that people with mental illness die prematurely and have higher rates of comorbidities than the general population [23, 24]. One reason for this may be “diagnostic overshadowing,” a process by which physical symptoms are misattributed to mental illness [25]. Patients with mental illness can present physical symptoms as behavioural changes resulting in diagnostic overshadowing, but they can also present mental discomfort as physical symptoms [26], which complicates the diagnostic assessment.

Risk of preventable harm could increase when a patient with high morbidity has more health care encounters and is therefore more exposed to health care. However, patients with psychiatric diagnoses seek health care later, resulting in more severe conditions by the time of diagnosis [21]. In the present study, the most common type of harm in this group of patients was diagnostic error, which should be less likely if the patient presents later in the disease course with more evident symptoms. Furthermore, if the patient has more health care encounters, the clinician would have more opportunities to correctly diagnose the patient.

Earlier studies have concluded that patients with a history of psychiatric disease have a significantly higher rate of early death after ED discharge than do patients in the ED without such a history. Most such patients die of non-psychiatric causes [27]. Previous studies have shown that patients with psychiatric diagnoses are at higher risk of patient safety events; however, these studies mostly included patients with schizophrenia and were conducted within United States hospital settings, a setting that may not be generalisable to European conditions [28–31]. In the present study, we included all psychiatric diagnoses in a European first-line health care context, thus increasing the external validity.

### Clinical implications

Health care need to approach the problem of increased risk for this group of patients systematically. In the hospital setting, the reasons for increased risk of harm in patients with psychiatric illnesses include difficulties of communication, different expressions of symptoms, problems in knowledge and information gathering, and substance misuse [25, 32]. The reasons for increased risk in primary care are not explored, but this group needs extra attention to mitigate that risk.

### Implications for future research

More research is needed to explore the reasons for the increased risk of harm to patients in first-line health care. In particular, very few studies within this area have been performed in PHC. Exploration of the ways in which care can be made safer for this vulnerable group should also be performed. Preferably, countermeasures to prevent harm should be designed in co-production with patients representing this group [33].

## Conclusion

Patients with a broad range of psychiatric illness are at higher risk of preventable harm in first-line health care. This underlines the importance of a raised awareness as well as the need for better decision support for patients as well as providers.

## Data Availability

The datasets used and/or analysed during the current study are available from the corresponding author on reasonable request.

## Abbreviations

CI: Confidence interval
ED: Emergency departments
ICD-10: International Classification of Diseases, 10th revision
IVO: Health and Social Care Inspectorate
LÖF: Landstingens Ömsesidiga Försäkringsbolag
OR: Odds ratio
PHC: Primary health care

## References

[1] Jha AK, Larizgoitia I, Audera-Lopez C, Prasopa-Plaizier N, Waters H, Bates DW (2013). The global burden of unsafe medical care: analytic modelling of observational studies. BMJ Qual Saf.

[2] James JT (2013). A new, evidence-based estimate of patient harms associated with hospital care. J Patient Safety.

[3] Nilsson L, Borgstedt-Risberg M, Soop M, Nylen U, Alenius C, Rutberg H (2018). Incidence of adverse events in Sweden during 2013-2016: a cohort study describing the implementation of a national trigger tool. BMJ Open.

[4] Panagioti M, Khan K, Keers RN, Abuzour A, Phipps D, Kontopantelis E, Bower P, Campbell S, Haneef R, Avery AJ (2019). Prevalence, severity, and nature of preventable patient harm across medical care settings: systematic review and meta-analysis. BMJ.

[5] Panesar SS, de Silva D, Carson-Stevens A, Cresswell KM, Salvilla SA, Slight SP, Javad S, Netuveli G, Larizgoitia I, Donaldson LJ (2016). How safe is primary care? A systematic review. BMJ Qual Saf.

[6] Slawomirski LAA, Klazinga N (2018). The economics of patient safety in primary and ambulatory care. OECD.

[7] Cooper A, Edwards A, Williams H, Evans HP, Avery A, Hibbert P, Makeham M, Sheikh A, J Donaldson L, Carson-Stevens A (2017). Sources of unsafe primary care for older adults: a mixed-methods analysis of patient safety incident reports. Age Ageing.

[8] Medicine Io (2015). National Academies of sciences E, medicine: improving diagnosis in health care.

[9] Ball J, Balogh E, Miller BT (2015). Improving diagnosis in health care.

[10] Fernholm R, Pukk Harenstam K, Wachtler C, Nilsson GH, Holzmann MJ, Carlsson AC (2019). Diagnostic errors reported in primary healthcare and emergency departments: a retrospective and descriptive cohort study of 4830 reported cases of preventable harm in Sweden. Eur J Gen Pract.

[11] Singh H, Schiff GD, Graber ML, Onakpoya I, Thompson MJ (2017). The global burden of diagnostic errors in primary care. BMJ Qual Saf.

[12] Singh H, Meyer AN, Thomas EJ (2014). The frequency of diagnostic errors in outpatient care: estimations from three large observational studies involving US adult populations. BMJ Qual Saf.

[13] Martinez Sanchez A, Campos RM (2011). Detection of prescribing related problems at the community pharmacy. Int J Clin Pharm.

[14] Marbella AM, Laud PW, Brasel KJ, Layde PM (2011). Patient risk factors for medical injury: a case-control study. BMJ Qual Saf.

[15] Naessens JM, Campbell CR, Shah N, Berg B, Lefante JJ, Williams AR, Culbertson R (2012). Effect of illness severity and comorbidity on patient safety and adverse events. Am J Med Qual.

[16] Rothschild JM, Bates DW, Leape LL (2000). Preventable medical injuries in older patients. Arch Intern Med.

[17] Sari AB, Cracknell A, Sheldon TA (2008). Incidence, preventability and consequences of adverse events in older people: results of a retrospective case-note review. Age Ageing.

[18] Piccardi C, Detollenaere J, Vanden Bussche P, Willems S (2018). Social disparities in patient safety in primary care: a systematic review. Int J Equity Health.

[19] Fernholm R, Pukk Härenstam K, Wachtler C, Nilsson GH, Holzmann MJ, Carlsson AC (2019). Diagnostic errors reported in primary healthcare and emergency departments: a retrospective and descriptive cohort study of 4830 reported cases of preventable harm in Sweden. Eur J Gen Pract.

[20] Thomas EJ, Brennan TA (2000). Incidence and types of preventable adverse events in elderly patients: population based review of medical records. BMJ.

[21] Somatic care and morbidity in cancer with existing co-morbidity of mental illness; 2013. [https://www.socialstyrelsen.se/globalassets/sharepoint-dokument/artikelkatalog/nationella-riktlinjer/2013-6-27.pdf].

[22] Pukk K, Lundberg J, Penaloza-Pesantes RV, Brommels M, Gaffney FA (2003). Do women simply complain more? National patient injury claims data show gender and age differences. Qual Manag Health Care.

[23] Saxena S (2018). Excess mortality among people with mental disorders: a public health priority. Lancet Public Health.

[24] Charlson FJ, Baxter AJ, Dua T, Degenhardt L, Whiteford HA, Vos T. Excess Mortality from Mental, Neurological, and Substance Use Disorders in the Global Burden of Disease Study 2010. In: Patel V, Chisholm D, Dua T, Laxminarayan R, Medina-Mora ME, editors. Mental, Neurological, and Substance Use Disorders: Disease Control Priorities, vol. 4. 3rd ed. Washington (DC); 2016. 27227239

[25] Jones S, Howard L, Thornicroft G (2008). ‘Diagnostic overshadowing’: worse physical health care for people with mental illness. Acta Psychiatr Scand.

[26] Wallerblad A, Moller J, Forsell Y (2012). Care-seeking pattern among persons with depression and anxiety: a population-based study in Sweden. Int J Family Med.

[27] Chang BP, Pany MJ, Obermeyer Z (2017). Early death after emergency department discharge in patients with psychiatric illness. Am J Emerg Med.

[28] Khaykin E, Ford DE, Pronovost PJ, Dixon L, Daumit GL (2010). National estimates of adverse events during nonpsychiatric hospitalizations for persons with schizophrenia. Gen Hosp Psychiatry.

[29] Daumit GL, McGinty EE, Pronovost P, Dixon LB, Guallar E, Ford DE, Cahoon EK, Boonyasai RT, Thompson D (2016). Patient safety events and harms during medical and surgical hospitalizations for persons with serious mental illness. Psychiatr Serv.

[30] McGinty EE, Thompson DA, Pronovost PJ, Dixon LB, Guallar E, Ford DE, Cahoon EK, Boonyasai R, Daumit GL (2017). Patient, provider, and system factors contributing to patient safety events during medical and surgical hospitalizations for persons with serious mental illness. J Nerv Ment Dis.

[31] Smith EG, Zhao S, Rosen AK (2012). Using the patient safety indicators to detect potential safety events among US veterans with psychotic disorders: clinical and research implications. Int J Qual Health Care.

[32] van Nieuwenhuizen A, Henderson C, Kassam A, Graham T, Murray J, Howard LM, Thornicroft G (2013). Emergency department staff views and experiences on diagnostic overshadowing related to people with mental illness. Epidemiol Psychiatr Sci.

[33] Lwembe S, Green SA, Chigwende J, Ojwang T, Dennis R (2017). Co-production as an approach to developing stakeholder partnerships to reduce mental health inequalities: an evaluation of a pilot service. Prim Health Care Res.

